# Myocardial Dysfunction in Sepsis: A Large, Unsolved Puzzle

**DOI:** 10.1155/2012/896430

**Published:** 2012-03-13

**Authors:** Constantino Jose Fernandes Jr., Murillo Santucci Cesar de Assuncao

**Affiliations:** ^1^Hospital Israelita Albert Einstein, Avenida Albert Einstein 627/701, 05661-901 Morumbi, SP, Brazil; ^2^Department of Intensive Care Unit, Hospital Israelita Albert Einstein, Sao Paulo, Brazil

## Abstract

Sepsis has high incidence and mortality rates around the world. The role of cardiac depression in myocardial dysfunction during sepsis remains to be elucidated. This review attempts to summarize our understanding of the anatomical, histopathological, and pathophysiological mechanisms behind cardiac dysfunction. Biomarkers to detect cardiac depression have been used to recognize developing problems, but the actual impact of these tools remains unclear.

## 1. Introduction

Sepsis and sepsis-induced mortality are major health concerns worldwide [1–3]. Septic shock is the most severe form of sepsis and is one of the most significant causes of death among critically ill patients. It is characterized by hemodynamic changes and the dysfunction of one or more organs. Septic shock is a kind of distributive shock, but it can contain components from other forms of shock, such as hypovolemic, cardiogenic, and obstructive shock, which can manifest in the form of profound hypovolemia, severe cardiac failure, or pulmonary arterial hypertension, respectively. Cardiovascular changes are important in septic shock; peripheral vascular dysfunction, which can result in heterogeneous microcirculatory flow, can frequently induce myocardial depression. In this population, cardiovascular collapse can increase the risk of death in sepsis as much as two times, and myocardial depression occurs in almost 40% of septic patients. Myocardial depression is characterized by a cardiac output that fails to meet metabolic demands [4, 5]. In a recent, elegant study, Vieillard-Baron et al. observed a reduced left ventricular ejection fraction (LVEF < 45%) in 60% of patients during the first 3 days of the treatment of septic shock. Curiously, 39% of these patients had presented with left ventricular hypokinesia at admission, suggesting that the development of LVEF can occur in the earliest sepsis stage [6]. In general, approximately 15% of the deaths related to septic shock are secondary to myocardial depression [5].

Myocardial dysfunction can determine whether a patient survives. In survivors, ventricular compliance is increased with a higher end-diastolic volume that helps maintain an adequate stroke volume. Nonsurvivors, in contrast, are progressively unable to maintain the same stroke volume because of reduced diastolic compliance [7]. Reversible myocardial depression also plays a role in survival. It is transient in survivors, typically with a limited duration of 7 to 10 days after the onset of sepsis. As the inflammatory response is attenuated, myocardial dysfunction decreases in a self-limiting manner. Supportive measures, such as the optimized preload and use of inotropes, can be critical for maintaining adequate blood flow to keep tissues perfused. Wiggers described the concept of reversible myocardial depression or dysfunction in 1947. He postulated the existence of a myocardial depressant factor responsible for myocardial dysfunction in hemorrhagic shock [8]. During the 1960s and 1970s, experimental studies showed evidence of transient myocardial dysfunction in several forms of disease, including hemorrhagic and septic shock [9].

It was previously postulated that decreased circulating blood volume was responsible for the reduced cardiac output observed in septic shock. More recent studies demonstrate that this assumption is particularly true before fluid resuscitation occurs, when there may be an imbalance between continent (vessels) and inside it. After volemic restoration, however, when cardiac output does not match oxygen demand and there is no recruited preload, a level of myocardial depression may still occur. Recent studies have shown that patients with septic shock who were adequately resuscitated typically displayed a high cardiac output and also displayed myocardial depression in the form of a low systemic resistance hemodynamic circulatory condition. It is estimated that only 10 to 20% of patients who have myocardial depression need to receive inotropic drugs [4].

## 2. Myocardial Depression: A Puzzle

The pathophysiology of cardiac depression is complex and involves a multitude of factors [10]. This review on the mechanisms of cardiac depression in sepsis will include anatomical, histopathological, and pathomechanistic data.

Many anatomopathologic alterations have been described in fatal cases of septic shock. In 1948, Moon described degenerative myocardial changes in 21 patients who developed shock due to trauma [11]. In a necropsy study, Fernandes Júnior et al. enrolled 10 septic shock patients and observed the presence of interstitial myocarditis, necrotizing vasculitis, and myocardial abscesses, demonstrating that the heart is affected by endotoxins, mediators, and sometimes the direct action of bacteria [12]. In 1994, the same authors published a histopathological analysis of the myocardium of 71 patients who were autopsied after meeting the morphological criteria for sepsis. The authors observed the presence of interstitial myocarditis in 27% of the patients, bacterial colonization in 11%, necrosis of cardiac fibers in 7%, and interstitial edema in 28%, although this last finding was not significantly different from the control group [13].

## 3. The First Piece of the Puzzle: Endotoxins

Endotoxins mediate cardiovascular changes that mimic sepsis in both research animals [14] and human volunteers [15]. Suffredini et al. evaluated the cardiovascular effects of endotoxemia by injecting nine healthy volunteers with a bolus dose of endotoxin. Three hours after the injection, a physiological response resembling severe sepsis was observed, characterized by an increased heart rate, high blood flow, and a reduction in systemic vascular resistance (SVR). After intravascular resuscitation, there was a reduction in LVEF and LV performance. Endotoxins can contribute to myocardial depression through interactions with the cell membrane receptor TLR-4, and in myocardial depression, endotoxins initiate inflammation through mediators such as cytokines, nitric oxide (NO), and C5 [16]. Despite these facts, a direct role for endotoxins in sepsis is in doubt because many septic patients do not have detectable endotoxin levels in their blood and because Gram-positive sepsis exists, which is clinically similar to Gram-negative sepsis but is independent of endotoxins [16].

## 4. Connecting the Pieces: Toll-Like Receptors

 An inflammatory immune response begins with the recognition of microorganisms and is mediated by pattern recognition receptors, such as the Toll-like receptor family (TLRs), which bind to highly conserved molecules on the pathogen called pathogen-associated molecular proteins (PAMPs). TLRs that recognize components of the bacterial cell wall are expressed on the cell membrane, while TLRs that recognize nucleic acids are located within the host cell. TLR1 recognizes Gram-positive bacteria, TLR2 recognizes the peptidoglycan of Gram-positive bacteria, and TLR4 binds the lipopolysaccharide of Gram-negative bacilli [16, 17].

In the heart, a number of infiltrating and resident immune cells express TLR4. As a consequence, the activation of TLR4 signaling pathways may directly cause myocyte dysfunction. Alternatively, the activation of TLR4 signaling pathways may result in the production of various mediators by leukocytes (interleukin-1 (IL-1) and tumor necrosis factor-alpha (TNF-*α*)), which may result in cardiac impairment. Both microbial and endogenous proinflammatory mediators, such as bacterial endotoxin (lipopolysaccharide (LPS)), IL-1, and TNF-*α*, may directly depress myocardial contraction [18, 19]. These factors appear to be important because mice deficient in TLR4 or IRAK1 (IL-1 receptor-associated kinases 1) are protected from LPS-induced mortality and cardiac dysfunction [20]. IRAK1 is a component of the TLR pathway, which triggers the activation of NF*κ*B and the transcription of proinflammatory cytokines and chemokines. A recent study exposed animals to either LPS challenge (septic shock model) or to coronary artery ligation (myocardial ischemia (MI) model) and found that TLR4-deficient mice challenged with LPS or MI displayed reduced cardiac function, increased myocardial levels of IL-1*β* and TNF-*α*, and the upregulation of mRNA encoding TLR4 prior to myocardial leukocyte infiltration [21].

## 5. Coronary Flow: No Contribution for the Puzzle

In 1986, Cunnion et al. disproved the idea that coronary hypoperfusion and ischemic phenomena could be involved in the myocardial dysfunction mechanism. In a study with coronary sinus catheterization, they demonstrated that coronary flow was the same or greater in septic shock patients when compared to normal individuals and that the production of lactic acid was normal [22]. This result eliminated ischemia as a major factor in sepsis-induced cardiac dysfunction.

## 6. Myocardial Depressant Factor: The Key Piece

In 1947, Wiggers [8] observed the presence of a myocardial depressant factor in an experimental model of hemorrhagic shock. In the 1960s, many authors described similar substances responsible for myocardial depression. In the mid-1970s, Lefer documented the existence of a myocardial depressant factor (MDF) in the blood of dogs during induced endotoxic shock and suggested that it was a peptide of 800 to 1,000 daltons that originated in the pancreas [23]. To demonstrate the existence of a myocardial-depressant substance, McConn infused the coronary ostia of dogs with the plasma of septic patients, demonstrating the presence of two molecules with depressor activity [24]. The first fraction, with a weight of less than 1 kDa, showed an immediate depressant effect, whereas the second fraction, with a weight between 1 and 10 kDa, showed late depressor activity.

Subsequent studies have proposed several substances as MDF including tumor necrosis factor-alpha (TNF-*α*), interleukin-1*β* (IL-1*β*), C5a, and endotoxin. All of these factors, except endotoxin, are intrinsic substances made by the immune and other systems; endotoxin, however, is an extrinsic factor released by the breakdown of Gram-negative bacteria [25].

The main inflammatory mediators that contribute to myocardial depression in sepsis include interleukins (IL-2, IL-4, IL-6, IL-8, and IL-10), gamma interferon (IFN-*γ*), TNF-*α*, IL-1*β*, and C5a. The action of IL-2 in septic shock has not yet been thoroughly determined; however, it probably mediates the release of TNF-*α* and IL-1. In patients undergoing IL-2 cancer immunotherapy, myocardial depression, myocarditis, and myocardial necrosis are observed. Despite having MDF characteristics, IL-4, IL-8, and IL-10 did not cause significant hemodynamic changes when injected in experimental models. Additionally, none of these cytokines induced myocardial depression when tested *in vitro*. IL-6 is more of a marker than a mediator of sepsis, and it is a good predictor of mortality in septic shock [26]. IFN-*γ* has mild depressor effects when it acts in isolation, but it acts synergistically with TNF-*α*, IL-1, and other inflammatory factors, enhancing their effects both *in vivo* and *in vitro* [27]. The two cytokines that show the greatest cardiovascular effects in animals and humans are TNF-*α* and IL-1*β*. When a small quantity of endotoxin was injected into humans, increased levels of TNF-*α* were noted [16], while the administration of recombinant TNF-*α* in animal models led to the appearance of fever, lactic acidosis, hemodynamic changes, and even death [20].

The most important inflammatory mediators in myocardial depression in sepsis are TNF-*α* and IL-1. In contrast to the other cytokines mentioned previously, they were shown to be involved in cardiac cell contraction when injected *in vitro* and observed by electronic microscopy. Additionally, TNF-*α* and IL-1 show the greatest cardiovascular effect in animals and humans [28]. Preincubation of live, neonatal rat cardiomyocytes with TNF-*α* blocks *β*-adrenoceptor-mediated increases in pulsation amplitude and mitochondrial oxygen consumption [29]. The administration of recombinant TNF-*α* in animal models led to the appearance of fever, lactic acidosis, hemodynamic changes, and even death. Many studies using anti-TNF-*α* antibodies in humans and other animals showed a rapid improvement in cardiovascular parameters, with no decrease in mortality [28, 30]. IL-1 consists of two distinct ligands (IL-1*α* and IL-1*β*) with high-sequence homology and indistinguishable biological activities, and both are synthesized as large precursor proteins. IL-1*α* remains intracellularly unless released by a dying cell, and it is kept in an active, Pro-IL-1*α* form, that is, cleaved by calpain to generate the mature protein. In contrast, pro-IL-1*β* is biologically inactive until it is enzymatically cleaved by caspase-1 to generate the active 17.5 kDa protein [31]. This cytokine also reproduced the hemodynamic effects found in septic shock when infused into animals. TNF-*α* and IL-1*β* depress human myocardial function, even at low doses, both in isolation and synergistically [19]. The maximum effect of TNF-*α* in TNF-*α*-challenged dogs was observed between 8 and 48 h after treatment [14], and it induced nitric oxide synthase (iNOS) and enhanced production of nitric oxide (NO) in the heart. The concept of TNF-*α*-induced cardiodepression is supported by the induction of iNOS and the inhibition of constitutive NOS (cNOS) or endothelial NOS (eNOS) at high TNF-*α* concentrations. This idea is also supported by NO-independent cardiodepression at low, pathophysiologically relevant concentrations. TNF-*α* promotes the release of sphingosine and the transient suppression of calcium [32]. Additionally, Finkel et al. demonstrated that TNF-*α*, IL-6, and IL-2 inhibited the contraction of isolated hamster papillary muscles in a concentration-dependent, reversible manner. When the NO synthase inhibitor NG-monomethyl-L-arginine (L-NMMA) was tested, it blocked these negative inotropic effects, and L-arginine reversed the inhibition by L-NMMA. These findings demonstrate that the direct, negative, inotropic effects of cytokines are mediated through myocardial nitric oxide synthase [33].

C5a, a protein fragment released from the complement component C5, has also been of interest in the pathogenesis of sepsis-induced cardiac depression. In a rat model of sepsis, *in vivo* cardiac depression was prevented by the administration of a blocking antibody against C5a. This experiment also showed increased C5a receptor levels in affected cardiomyocytes, suggesting a promising approach for preventing and treating sepsis-induced cardiac depression that may be used in future human studies [34].

## 7. Another Piece of the Puzzle: Nitric Oxide (NO)

 NO is synthesized upon the cleavage of L-arginine to L-citrulline by three distinct isoforms of NO synthase (NOS) within the myocardium: neuronal NO synthase (NOS1), inducible NOS (NOS2), and endothelial NOS (NOS3) [35]. NOS1 and NOS3 are constitutively expressed in cardiac myocytes and produce NO in conjunction with myocyte contraction due to Ca-calmodulin regulation. As discussed previously, NO can be released by cytokine stimulation via NOS2. This molecule is only expressed during inflammatory responses and is present during many pathophysiological conditions other than sepsis, including ischemia-reperfusion injury and heart failure. When expressed, NOS2 produces much higher levels of NO independent of [Ca^2+^]*_i_*, as compared to the constitutive NOS isoforms [36], and it is an important mediator in sepsis. The impact of NO on cardiac function is complex because it has an effect on systemic vascular tone, thus impacting preload, afterload, and coronary vascular tone. NO signals through at least two distinct pathways: cyclic guanosine monophosphate- (cGMP-) dependent and cGMP-independent pathways. The cGMP-dependent effects of NO result from the NO-induced activation of guanylate cyclase, leading to increased cGMP levels that modulate the activity of protein kinase G (PKG) and cGMP-regulated phosphodiesterases (PDE; cGMP stimulated: PDE2; cGMP inhibited: PDE3). cGMP-independent effects occur mainly via S-nitrosylation, an important protein modification related to cell signaling [36]. This multifaceted involvement of NO in cardiac physiology is supported by a tight molecular regulation of the three NO synthases, from cellular spatial confinement to posttranslational allosteric modulation, by specific interacting proteins acting in concert to restrict the influence of NO to a particular intracellular target in a stimulus-specific manner.

NOS3 is present in endothelial cells, platelets, the brain, and the myocardium and is dependent on calcium. In the myocardium, NOS3 expression appears to be graded, such that NOS3 expression in left ventricular epicardial myocytes is significantly increased compared to left ventricular endocardial myocytes. Myocardial NOS3 may have an important protective role against sepsis-induced myocardial dysfunction. Experiments have shown that mice with cardiomyocyte-specific NOS3 overexpression are protected from myocardial dysfunction and death associated with endotoxemia. Increased myocardial NO levels attenuate endotoxin-induced reactive oxygen species production and increase total sarcoplasmic reticulum Ca^2+^ levels and myofilament sensitivity to Ca^2+^ [37]. In a recent study, Bougaki et al. compared wild-type (WT) and NOS3-deficient (NOS3KO) mice submitted to severe polymicrobial sepsis induced by colon ascendens stent peritonitis. They found that cardiac output was markedly depressed only in NOS3KO mice but not in WT mice. These data also suggested that NOS3 protects against systemic inflammation and myocardial dysfunction after peritonitis-induced polymicrobial sepsis in mice [38]. NOS1 is present in the central and peripheral nervous system. Moreover, NOS1 is also constitutively expressed in cardiac myocytes. Several studies have shown that NOS1 is capable of regulating the *β*-adrenergic receptor (*β*-AR) pathway. In particular, *in vivo* and whole heart experiments demonstrated that the knockout of NOS1 leads to a reduced contractile response to *β*-AR stimulation [39].

NOS2 can be found in macrophages, neutrophils, Kupffer cells, and hepatocytes, and it does not depend on calcium. While low doses of NO may increase LV function, excessive NO delivery from inflammatory cells (or cytokine-stimulated cardiomyocytes themselves) may result in profound cellular disturbances that lead to heart failure [40]. Echocardiographic examination of the cardiac function of wild-type and NOS2-deficient mice after the infusion of endotoxin demonstrated preserved myocardial performance in the NOS2-deficient group [41]. Under normal conditions in the vascular endothelium, NOS1 converts L-arginine into NO via calcium and NADPH in response to endothelial stimulation caused by stress or mediators of vasodilation, such as acetylcholine, bradykinin, or histamine. NO has a short half-life (between 6 and 10 seconds) but displays a great diffusion potential. It enters the cytosol of the adjacent smooth muscle cell where it activates soluble guanylate cyclase to produce cGMP, which in turn promotes the sequestration of calcium in the sarcoplasmic reticulum through L-type calcium channels. Cytoplasmic calcium then diminishes, leading to smooth muscle relaxation and consequent vasodilation [33]. This process also occurs in cardiac cells, resulting in decreased myocyte contraction. Balligand et al. examined the interplay of bacterial endotoxin, macrophage stimulation, NOS2, cGMP production, and reduced contractility. In this study, rat macrophages were stimulated by exposure to LPS and were then incubated with rat cardiomyocytes for 24 h. The amplitude of myocytes stimulated by *β*-agonists was significantly reduced in LPS-treated cells relative to controls, and this reduction was abolished by the addition of the NOS inhibitor L-NMMA. Additionally, LPS exposure increased both nitrite production in myocytes and cGMP formation in fibroblasts, suggesting a possible cGMP-mediated mechanism for NO-induced negative inotropy [42]. Recent evidence indicates that most of the cytotoxicity attributed to NO is actually due to peroxynitrite produced from the diffusion-controlled reaction between NO and another free radical, the superoxide anion. Peroxynitrite interacts with lipids, DNA, and proteins via direct oxidative reactions or indirect methods and can be highly cytotoxic [43].

## 8. Endothelin 1

Endothelin 1 affects myocardial contractile properties and the progression of myocardial hypertrophy. Elevated concentrations of ET-1 in both the plasma and myocardium have been observed during sepsis and endotoxemia. However, the role of elevated ET-1 during sepsis is not completely understood. During endotoxemia, the inhibition of ET-1 biosynthesis leads to the downregulation of p38Y mitogen-activated protein kinase (p38-MAPK) phosphorylation and the expression of NO synthase II [27]. Sharma et al. showed that the induction of sepsis produced a biphasic response until 48 h (elevated concentration of ET-1 at 4, 8, and 12 h returned to baseline values at 24 and 48 h), but this induction was associated with depressed myocardial performance [44]. Chopra and Sharma found that the elevation of ET-1 at 3 and 7 days postsepsis correlated with depressed cardiodynamics [45]. This result suggests that the profile of ET-1 has a triphasic response, an initial peak at 4 to 12 h, and then a second peak at 3 to 7 days postsepsis. Because the second peak of ET-1 is associated with depressed myocardial contractility, these researchers speculate that the elevation of ET-1 during late sepsis could be detrimental to myocardial function.

## 9. One More Piece: The Contribution of Oxidative Stress

Reactive oxygen species (ROS) production is important for normal cellular function and survival. Oxidative stress results when ROS production and antioxidant protection mechanisms are unbalanced. Endotoxins can induce superoxide production via xanthine oxidase, NADH/NADPH oxidases, and mitochondria. It remains unclear if the self-amplifying cycle of ROS generation and mitochondrial damage occurs with mitochondrial dysfunction leading to oxidative stress and more mitochondrial impairment as the primary event, or if oxidative stress initiates mitochondrial dysfunction and further ROS release [46]. In cardiomyocytes, ROS generation in endotoxin-treated hearts is associated with impaired cardiac contraction and oxygen consumption. Activated neutrophils are the main source of ROS production, but a portion of the superoxide produced in the septic myocardium is derived from activated mononuclear cells. One of the key sources of superoxide in mononuclear cells is NADH and/or NADPH oxidase. This inducible electron transport system transfers reducing equivalents from NADH or NADPH to oxygen, resulting in superoxide anion (O_2_^−^) generation, and it is activated by endotoxins in both neutrophils and cardiomyocytes [47]. Overproduction of prostanoids may also be involved in the myocardial dysfunction associated with sepsis. This overproduction occurs by the inducible isoform of cyclooxygenase (COX-2) via activation of extracellular signal-regulated kinases (ERK1/2), p38 kinases, and c-Jun NH2-terminal kinases (JNK1/2/3) [48].

Oxidative stress-mediated mitochondrial damage, therefore, appears to be fundamental to the pathophysiology of organ failure in sepsis, suggesting a therapeutic role for antioxidants. Mitochondrial damage is mainly the result of an inhibition of electron flow through Complexes I, III, and/or IV. Administration of a superoxide scavenger compound prevented the mitochondrial abnormalities and improved cardiac contractile function in an animal model of endotoxemia. Targeting antioxidants to mitochondria may offer a novel therapy in the future, but clearly further studies are needed [46].

## 10. Autonomic Dysregulation and Calcium Flux

 During sepsis, a reduction in heart rate variability is a measure of autonomic dysregulation that reflects a loss of the balance between the sympathetic and vagal tone. Not only is the reduction in heart rate variability long lasting in patients with MODS, but also baroreflex sensitivity and chemoreflex sensitivity are impaired. These dysfunctions correlate with an unfavorable prognosis [49]. However, the correlation between the severity of autonomic dysfunction and the severity of septic cardiomyopathy remains unknown. Another important observation is that levels of catecholamines are elevated in sepsis models. There was a decreased density of *β*-AR on the myocardium in a murine model of sepsis, and this decrease was associated with a disruption of myocardial signal transduction after *β*-AR stimulation that includes decreased levels of stimulatory G-proteins and increased expression of inhibitory G-proteins [50, 51]. This trend was also noted in the myocardium of human nonsurvivors of septic shock. Zorn-Pauly et al. isolated human myocytes from right atrial appendages and incubated them for 6 to 10 h with LPS to investigate the pacemaker current *I*_*f*_. They observed that LPS-induced *I*_*f*_ impairment reduced the responsiveness of the model cell to fluctuations of autonomic input, showing a direct impact of LPS on the cardiac pacemaker current *I*_*f*_. This impact may contribute to the clinically observed reduction in heart rate variability in septic patients [52]. The myocardial depression in sepsis may be attributed to a desensitization of *β*-ARs due to an excess of catecholamines and to endotoxin action on the effects of the cardiac pacemaker current *I*_*f*_ on ionic channels.

Several studies have explored calcium involvement in the myocardial depression associated with sepsis. Calcium has a crucial and important role in myocardial contraction. All contractile pathways involving calcium can be connected to myocardial dysfunction. Several studies demonstrated that calcium has reduced peak currents during endotoxemia [53, 54]. Impaired calcium uptake, impaired release from calcium sarcoplasmic reticulum storage, and decreased calcium channel sensitivity are all involved in sepsis-related cardiac depression [55, 56].

## 11. Myocardial Depression in a Clinical View

Biomarkers such as cardiac troponin T and I have also been studied in sepsis. Elevated cardiac troponin T and I levels correlate with the presence of left ventricular systolic dysfunction [57]. Experimental evidence supports the view that troponin leakage is possible even if myocardial necrosis does not occur. Piper et al. demonstrated reversible membranous bleb formation in rat cardiomyocytes during limited periods of hypoxia and the concurrent release of myocardial enzymes into the cell supernatant [58]. In addition, in a study by Ver Elst et al., histopathologic examination revealed contraction band necrosis in only half of patients with a positive, premortem troponin level, and in one troponin-negative patient, suggesting that troponin release does not necessarily indicate myocardial cell necrosis [59]. Furthermore, levels of cardiac troponin also correlate with the duration of hypotension and the intensity of vasopressor support in patients with septic shock. Turner et al. measured cardiac troponin I levels in patients with septic shock and revealed evidence of ongoing structural myocardial cell injury during the course of severe sepsis and septic shock [60]. It is not clear whether troponin I levels reveal a role for cardiac injury in myocardial dysfunction or whether the injury is a result of other factors, including inflammatory mediators or exogenous catecholamine administration. In a study of 37 patients with septic shock, 16 (43%) patients with elevated serum cTnI had a significantly lower EF and a significantly higher mortality rate. A significant correlation between the serum level of cTnI and the reduction in EF was also observed [61]. In another study of 46 patients with septic shock, increased plasma concentrations of cTnI and cTnT were found in 50% and 36% of patients, respectively. LV functional assessment by two-dimensional TOE revealed that both cTnI and cTnT were exclusively associated with LV dysfunction (*P* < 0.0001) [59]. The potential role of B-type natriuretic peptide (BNP) as a biomarker has also been evaluated in septic patients. Recent studies have shown an increase in BNP levels in patients with severe sepsis and septic shock [62, 63]. BNP levels correlate with the degree of myocardial dysfunction and mortality. Rivers et al. found elevated BNP levels (>230 pg/mL) associated with myocardial dysfunction, and severity of global tissue hypoxia. In this population study, when adjusted for age, gender, history of heart failure, renal function, organ dysfunction and mean arterial pressure, a BNP greater than 210 pg/mL at 24 h was the most significant independent indicator of increased mortality [62]. In a smaller study, Kandil et al. observed that patients with septic shock had significantly higher BNP levels upon admission in comparison to a control group. Indeed, these data positively correlated with Sequential Organ Failure Assessment scores (*r* ^2^ = 0.74, *P* < 0.05) and prognosticated survival [63]. Turner et al. found a relationship in surgical septic patients between BNP levels and sepsis severity with early systolic dysfunction, which in turn is associated with death. In this study, a low ejection fraction (EF) (<50%) was associated with higher BNP (by Fisher's exact test; *P* < 0.05), and patients with low ejection fractions had a higher mortality (low EF 39% versus normal EF 20%; odds ratio = 3.03) [64]. In a recent prospective, observational, multicenter cohort study in 10 emergency departments, Perman et al. enrolled 825 patients to evaluate a composite of in-hospital mortality, severe sepsis, or septic shock within 30 days following presentation. The area under the curve (AUC) for BNP to predict the triple composite outcome was 0.69, and the optimal cutoff point of BNP was 49 pg/mL. Patients with a BNP > 49 pg/mL had a greater mortality rate (*P* = 0.0001), a greater risk of development of severe sepsis (*P* = 0.0001) and septic shock (*P* = 0.0001), and a higher rate of the triple composite outcome (unadjusted odds ratio [OR] = 1.9, 95% confidence interval [CI] = 1.6 to 2.1; *P* < 0.001). The sensitivity was 63% (95% CI = 58% to 67%), the specificity was 69% (95% CI = 65% to 73%), the negative predictive value (NPV) was 63% (95% CI = 58% to 67%), and the positive predictive value (PPV) was 69% (95% CI = 65% to 74%). In multivariate analysis, after adjusting for age, gender, heart rate, white blood cell count, and creatinine, an elevated BNP was associated with increased odds of having a composite outcome. Outcome was similar in patients who did not have severe sepsis or septic shock upon arrival. BNP levels can be useful in prognosis but have some limitations [65]. In patients with severe sepsis and septic shock, elevated BNP levels are associated with organ and myocardial dysfunction, global tissue hypoxia, and mortality. All biomarkers can help in identifying high-risk patients, but there still remains doubt as to whether they can be utilized as organ dysfunction parameters, severe illness criteria, or even as treatment guides. The best way to utilize these biomarkers in clinical practice remains unsolved.

Bouhemad et al. performed a prospective echocardiographic study to assess changes of left ventricular dimensions over time in patients with septic shock, and they associated those changes with troponin levels. They identified two groups of troponin I- (cTnI-) positive septic patients whose clinical and echocardiographic features were markedly different. Among patients with increased cTnI levels, patients could be separated according to echocardiographic left systolic ventricular dysfunction or isolated impairment of left ventricular relaxation. The concept of preload recruitment applies exclusively to patients with systolic left ventricular impairment, where ventricular enlargement may represent an adaptive mechanism to maintain cardiac output. Those patients presenting with ventricular relaxation impairment despite showing no ventricular dilation or a drop in ejection fraction exhibited the worst prognosis [66].

## 12. Treatment Options

The best treatment for myocardial dysfunction is the proper management of sepsis. The early collection of hemocultures in conjunction with adequate antibiotic care is the gold standard. Moreover, aggressive fluid replacement to remedy hypovolemia, guided by the examination of fluid responsiveness parameters, appears to be a rational strategy. This approach aims to provide adequate perfusion, as evaluated by central venous saturation (SvcO_2_) optimization and lactate clearance. Keeping arterial pressure stable is very important for reestablishing organ perfusion pressure, which helps maintain blood flow to tissues. Norepinephrine is the vasopressor of choice when a patient is nonresponsive to fluids.

As cited above, only 10 to 20% of patients who have myocardial depression will need to receive inotropic drugs to obtain adequate tissue perfusion. Most patients will benefit from administration fluid infusion. However, when inotropes are indicated to optimize flow and cardiac output and improve hemodynamics, dobutamine is the first choice. Patients may have a poor response to *β*-adrenergics due to myocardial depression. In this situation, an alternative is levosimendan, a calcium sensitizer, which in some clinical and experimental studies has improved perfusion [67–69]. Only one prospective randomized controlled trial has compared the effects of levosimendan and dobutamine. This small trial enrolled 28 septic shock patients with LV dysfunction (LVEF < 45%) persisting after 48 h of conventional treatment. The patients were then randomized to receive a 24-h infusion of either levosimendan (0.2 *μ*g^−1^·kg^−1^·min^−1^) or dobutamine (5 *μ*g^−1^·kg^−1^·min^−1^). Mean arterial pressure was sustained at approximately 70–80 mmHg with norepinephrine and volume therapy, which was guided by a Swan-Ganz catheter. Systemic or regional hemodynamic variables were evaluated. Although dobutamine did not alter any of the studied parameters, levosimendan had beneficial effects on both cardiovascular performance and regional perfusion. Indeed, the use of levosimendan was associated with a reduction in LV end-diastolic volume and a significant increase in the stroke index, the cardiac index, the oxygen delivery index, the oxygen consumption index, and the left ventricular stroke work index. Levosimendan also resulted in increased gastric mucosal flow, creatinine clearance, and urinary output and decreased lactate concentrations by lowering Gap PCO_2_ and improving lactate clearance and SvO_2_ [67]. Unfortunately, no definitive studies support levosimendan as the best choice for patients presenting with myocardial dysfunction by sepsis.

## 13. Conclusions

Many questions concerning myocardial dysfunction in sepsis remain, spanning topics from pathomechanisms to treatment. In reality, only support treatment is available for septic patients, and no specific drugs can reverse this dysfunction. In the future, with new approaches in sepsis treatment and a better understanding of the mechanisms of disease, myocardial dysfunction will be improved.
